# Crosslinking in Semi-Batch Seeded Emulsion Polymerization: Effect of Linear and Non-Linear Monomer Feeding Rate Profiles on Gel Formation

**DOI:** 10.3390/polym13040596

**Published:** 2021-02-17

**Authors:** Chang Liu, Amit K. Tripathi, Wei Gao, John G. Tsavalas

**Affiliations:** 1 Department of Chemistry, University of New Hampshire, Durham, NH 03824, USA; cl1089@wildcats.unh.edu (C.L.); amit.tripathi@unh.edu (A.K.T.); 2 The Dow Chemical Company, Core R&D, Analytical Science, Collegeville, PA 19426, USA; weigao@dow.com

**Keywords:** crosslinking, semi-batch, seeded emulsion polymerization, cyclization, gel fraction, latex, non-linear feed

## Abstract

Waterborne latex is often called a product-of-process. Here, the effect of semi-batch monomer feed rate on the kinetics and gel formation in seeded emulsion polymerization was investigated for the copolymerization of n-butyl methacrylate (*n*-BMA) and ethylene glycol dimethacrylate (EGDMA). Strikingly, the gel fraction was observed to be significantly influenced by monomer feed rate, even while most of the experiments were performed under so-called starve-fed conditions. More flooded conditions from faster monomer feed rates, including seeded batch reactions, counterintuitively resulted in significantly higher gel fraction. Chain transfer to polymer was intentionally suppressed here via monomer selection so as to focus mechanistic insights to relate only to the influence of a divinyl monomer, as opposed to being clouded by contributions to topology from long chain branching. Simulations revealed that the dominant influence on this phenomenon was the sensitivity of primary intramolecular cyclization to the instantaneous unreacted monomer concentration, which is directly impacted by monomer feed rate. The rate constant for cyclization for these conditions was determined to be first order and 4000 s^−1^, approximately 4 times that typically observed for backbiting in acrylates. This concept has been explored previously for bulk and solution polymerizations, but not for emulsified reaction environments and especially for the very low mole fraction divinyl monomer. In addition, while gel fraction could be dramatically manipulated by variations in linear monomer feed rates, it could be markedly enhanced by leveraging non-linear feed profiles built from combination sequences of flooded and starved conditions. For a 2 h total feed time, a fully linear profile resulted in 30% gel while a corresponding non-linear profile with an early fast-feed segment resulted in 80% gel.

## 1. Introduction

Waterborne resins with low VOC (volatile organic compound) content are commonly produced by seeded emulsion polymerization. A key advantage of this reaction environment is the ability to achieve high molecular weight polymer while also maintaining tunable control over copolymer compositional uniformity, efficient heat removal during reaction, and the lack of a need for non-reactive solvent. Beyond the water present [1,2], the monomer itself can serve a second purpose as a plasticizer as a function of monomer feed rate to the reactor. As such, care must be taken into account for other potential impacts of process strategy on the balance of available reaction mechanisms. Crosslinking during polymerization to achieve a target network topology is, in fact, quite sensitive to process conditions, with macromolecular architecture a key design property in applications such as dental materials, paints and coatings, and adhesives [3,4,5,6]. For multiphase polymers, such as latex, strategies to tune polymer properties must consider multiple length scales from the individual chains, through to a large ensemble of chains in the form of a structured particle morphology, and finally in end-use application macroscopically. When gel content and macromolecular network characteristics are design parameters, it can also be important to control the extent and balance of intramolecular versus intermolecular crosslinking. For the case of latex particles that form from emulsion polymerization, intra- versus inter- also has a particle-scale definition, where separate from the molecular scale reactions we can consider a network within individual particles to be another form of “intra” and during film formation to a macroscopic contiguous polymer we can then describe particle–particle coalescence as “inter” [7,8,9]. The network within particles usually forms during the polymerization while the inter-particle events typically occur post-polymerization. In this scenario, one must consider that inter-particle coalescence can also be ineffective should the individual particle crosslink density and gel content be excessive, restricting interparticle diffusion of polymer chains. With the complexity of the heterogeneous reaction environment, and search for simultaneous tunable control of multiple property variables, a mechanistic understanding of both molecular scale chemistry choices in the system as well as the impact of reaction process conditions is critical. In certain cases, such challenges cannot be met by chemistry choices alone.

It is well known that waterborne latex is a product of processing. This is one of the key discussion points within the field of polymer reaction engineering [10]. There remain debates in the field on how best to interpret dominant mechanisms to gel content across datasets consistently [11,12,13,14], partially due to competing reactions to gel for acrylate-based systems [11,13] and also because the effect of processing was not always specifically isolated (comonomer selections were often simultaneously varied). Methacrylate systems copolymerized with dimethacrylates, which do not undergo chain transfer to polymers, are a very common monomer combination for a wide variety of applications, yet this combination has not yet been systematically studied for seeded emulsion polymerization; especially for low divinyl content.

Proper characterization of gel fraction in latex suffers its own challenges. Solvent extraction techniques remain the most widely used, yet for polymer colloids with diameters in the order of 100 nm decoupling soluble and insoluble fraction in a latex can be complex when filtration is involved, with mismatch in individual particle versus filter mesh size. In order to gain confidence in our in-house analysis of gel content collected from a solvent-extraction followed by centrifugation method, analysis from the newly established asymmetric flow field-flow fractionation (A4F) method coupled with multi-angle light scattering (MALS) [15,16] was cross-compared. Instead of defining gel based on solubility, A4F characterizes the total molecular weight distribution and assesses gel based on the high-end of the total molecular weight distribution, above the range that typical solvent-based techniques can handle (including standard gel permeation chromatography). Not only is A4F more direct than solvent-extraction based techniques, it also enjoys fewer systematic errors with less operator-specific choices and steps thus leading to higher precision and tighter confidence level. Even with this advantage, we were quite encouraged that our A4F results and trends on the crosslinked latex samples, with variation in monomer feed rate, were in excellent agreement with our centrifugation method employed post solvent-extraction of soluble fraction. This not only gave us confidence in the analytical results, but more so in the interpretations from the trends in the network topology versus monomer feed rate.

Given that, the objective of this work is to isolate and study the effect of processing conditions on network topology development within latex particles and to provide a general guideline for how to control gel content by varying the monomer feeding profile for methacrylate/dimethacrylate systems. To achieve this goal, a series of simple seeded emulsion copolymerizations of *n*-butyl methacrylate (*n*-BMA) and ethylene glycol dimethacrylate (EGDMA) with variations in monomer feeding profile were applied. We have previously described key elements of crosslinking efficiency and mechanism for high monomer concentration environments, such as in bulk polymerization, that focus on chemical choices (i.e., monomer structural features for both mono and divinyl contributors) [17,18]. Under those high monomer concentration conditions, the process mechanisms described here are almost inconsequential and masked by the chemical structural contributions. It should be emphasized that the region of the reaction space we are focusing on is low (less than 5%) divinyl content; the balance between these mechanisms may shift with higher divinyl content. Here in seeded emulsion polymerization, the reaction environment is highly viscous from the onset (as we start with a significant ratio of polymer seed to unreacted monomer). These conditions, quite different than bulk polymerization, can also generate significant gel content, yet are quite sensitive for the same chemical constituents to the monomer feed rate, with faster feeding tending to generate higher gel. We have verified this both experimentally and computationally where we observe that the impact of intramolecular primary cyclization of the divinyl chain-end radical to its own pendent vinyl is the dominant pathway impacting gel content for seeded emulsion polymerization, especially under starve-fed monomer feed conditions.

An exciting additional insight we emphasize here is that non-linear monomer feed profiles observed significantly higher gel content over the same total feed time compared to linear analogs. Non-linear feed profiles are commonly employed in industrial settings to avoid compositional drift, overcome heat removal issues, and enable or disable diffusion dynamically during portions of the polymerization, for example in multiphase morphology development. Here, we explored three different non-linear feeding profiles, each being combinations of three stages of either a flooded or a starved linear feeding profile. The results clearly showed that the gel content generated by non-linear feeding rates cannot be predicted by simple linear addition of gel contents generated by their parent linear feeding rates. This, we emphasize, is derived from the chronology of gel evolution during the polymerization. Early onset of any network polymer has dramatic impact on the probability for new gel to form in subsequent steps. As such, non-linear feed profiles can enable such a condition early in the polymerization and result in higher final gel content within the same total reaction time.

## 2. Materials and Methods

### 2.1. Materials

Monomers *n*-butyl methacrylate (*n*-BMA) and ethylene glycol dimethacrylate (EGDMA) were purchased from Acros Organics (New Jersey, NJ, USA). Inhibitor in the *n*-BMA was first removed by passing the monomer through a column packed with alumina (Fisher Scientific, Massachusetts, MA, USA). Potassium persulfate (KPS), ethyl acetate (EAc) and sodium dodecyl sulfate (SDS) were purchased from Sigma Aldrich (Missouri, MO, USA) as initiator, surfactant, and inert solvent for swelling latex, each used as received.

### 2.2. Synthesis of Seed Particles

Seed latex particles were prepared by a 2-step seeded emulsion polymerization growth after the synthesis of the primary seed by ab-initio emulsion polymerization. The recipe for the ab-initio emulsion polymerization is shown in Table 1. Before the reaction, SDS and water were added into a 1L jacketed reactor and heated to 70 °C under the purge of nitrogen. After mixing SDS and water for 15 min, KPS was added and mixed for 15 min, followed by 10 wt% of the total *n*-BMA added as a shot to generate the primary seed particles over 30 min. The remaining monomer was fed through a syringe pump over 3 h. After the monomer feed, the reactor was held at reaction temperature for 2 additional hours to complete monomer conversion.

The recipe for the first step growth of the primary seed is shown as Table 2. Here, 172.1 g of the first seed latex (22% solids) was utilized to seed this first semi-batch growth reaction. The reaction was again performed at 70 °C, in a 1 L jacketed reactor, under nitrogen blanket. Before polymerization, KPS and 1 g of SDS was dissolved within a portion of the water initial charge. SDS, seed latex and the remaining water were added to the reactor and mixed for 15 min. The KPS initiator, dissolved in a portion of the water, was then added and mixed for another 15 min, followed by the start of the *n*-BMA monomer feed. The total feeding time was 4.5 h with holding time after feeding at reaction temperature for 2 h. During the reaction, a second shot of SDS was dissolved with 15 g additional water and added to the reactor at 75 min after the start of the polymerization to account for the surface area growth. A third shot of SDS was also dissolved with 15 g additional water and added to the reactor at 180 min after the start of the reaction.

The second particle growth was done in the same manner. All crosslinking reactions were performed using the latex result of this second particle growth.

### 2.3. Seeded Emulsion Polymerization with Ethylene Glycol Dimethacrylate (EGDMA)

Typically, the 170 nm poly(*n*-BMA) latex, was used to seed a final growth reaction in a 3:1 mass ratio (poly(*n*-BMA-co-EGDMA) second stage at 1 w% EGDMA to poly(*n*-BMA) seed) targeting a final 265 nm particle diameter by seeded emulsion polymerization at 70 °C with variations in monomer feed rate. Feed times employed were 0 h (batchwise reaction), 0.5 h, 1 h, 1.5 h and 2 h, respectively, all for the same mass of monomer. After feeding, the reaction was held at 70 °C until a total reaction time of 3 h was achieved. Typically, experiments utilized a KPS concentration of 0.01 M with SDS as stabilizer.

Samples were taken periodically from the reactor for monitoring the dynamics of kinetics and gel formation development during the seeded emulsion co-polymerization with divinyl monomer present. The sampling intervals were varied based on feeding time and samples were quenched with hydroquinone.

### 2.4. Characterization

#### 2.4.1. Latex Particle Size Characterization

Particle size for each latex was measured by capillary hydrodynamic fractionation (CHDF) with a CHDF 2000 from Matec Applied Sciences (Massachusetts, MA, USA). The velocity gradients of fluid flow in the capillary were used to fractionate particles based on their particle sizes. Fractionated particles were quantified by an ultraviolet (UV) detector. The elution time for the latex samples was compared with the elution time for a marker solution (aqueous sodium benzoate solution). The time difference between both particle elution times along with a calibration curve was used to estimate to particle size. The CHDF was calibrated by 81 nm, 125 nm, 203 nm and 345 nm poly(styrene) standard latex particles (Thermo Scientific, Massachusetts, MA, USA)). Typically, a latex sample for CHDF measurement was prepared by diluting the original latex product via 3 drops of as-prepared latex added to a 2 mL vial which was then filled with deionized water.

#### 2.4.2. Gel Test by Solvent Extraction and Centrifugation

The dry latex sample (approximately 0.7 g) was immersed into acetone (8.5 g) in a centrifuge tube for 24 h. Acetone was chosen for the solvent as it is a good solvent for poly(*n*-BMA) as well as having a low density which helps facilitate the centrifugation process [19]. A latex sample with gel content will form a cloudy solution. After solubilizing the linear and lightly branched polymer content, the cloudy sample was centrifuged at 14,000× *g* at 15 °C for 30 min. After centrifugation, the supernatant (which contains the soluble portion of the polymer sample) was removed and the insoluble sedimented polymer was collected as gel and dried in an oven at 70 °C overnight. The gel fraction of the sample is estimated as:

(1) $$Gel\;Fraction=\frac{W_{gel}}{W_{dried}}\left(1+\frac{1}{SR_{t}\times X_{t}}\right)$$

where $W_{gel}$ is weight of the dried insoluble polymer, $W_{dried}$ weight of dry latex, $X_{t}$ conversion at time *t* and $SR_{t}$ the amount of monomer fed at time *t* per weight of seed polymer. It should be noted that the seed polymer does not contain any gel and the gel fraction is based on only the last growth stage polymer which includes the crosslinker. $X_{t}$, monomer conversion was characterized gravimetrically.

#### 2.4.3. Gel Characterization by Asymmetric Flow Field-Flow Fractionation Multiangle Light Scattering (A4F-MALS)

An A4F separation system was utilized for a comparative analysis of the total molecular weight distribution of the crosslinked polymers to assess gel fraction. Flows were delivered with an Agilent Technologies (California, CA, USA) 1200 series isocratic pump equipped with a microvacuum degasser. The AF4 flow regulation was controlled by an Eclipse 3+ system (Wyatt Technology, California, CA, USA). The separation channel dimensions were 15.2 cm in length and between 0.3 cm and 2.15 cm in width with a 350 µm-thick spacer. For all experiments, the membranes used were a Nadir regenerated cellulose membrane with a 5 kDa molecular weight cutoff (Wyatt Technology, California, CA, USA). All injections were performed with an autosampler (Agilent Technologies 1200 series, California, CA, USA). A multiangle light scattering (MALS) detector (DAWNHELIOS II, Wyatt Technology, California, CA, USA), and a differential refractometer (dRI, Optilab rEX, Wyatt Technology, California, CA, USA) were connected in series to characterize the fractions eluting from the AF4 channel. Data were collected and processed using Astra 7 software (Wyatt Technology, California, CA, USA).

The latex samples were prepared in ethyl acetate and filtered with one micrometer pore size syringe top filter before fractionation in ethyl acetate with A4F. The total gel content of each sample was calculated based on the dRI signal after baseline subtraction by a blank injection. The percent peak area of the gel peak is considered as total gel content. The weight percent gel content of the (second stage) polymer was adjusted to account for the non-crosslinked seed content.

## 3. Results and Discussion

### 3.1. Justification of Range of Monomer Feed Rates via Homopolymerization of n-Butyl Methacrylate (n-BMA) by Seeded Emulsion Polymerization

As our primary focus is to assess the impact and sensitivity of gel network development to monomer feed rate, we must first determine the tolerance of the general backbone polymerization to that monomer consumption versus delivery balance. Specifically, we must identify what range of feed times, for a constant monomer mass, would be classified as starve-fed or instead as flooded. Here, we define starve-fed conditions to correspond to systems where the instantaneous unreacted monomer concentration is at or below 1 mol/L (with reference to particle volume) throughout the polymerization. This low level of unreacted monomer not only minimizes plasticization of the seed polymer by monomer, but it also tends to lead to more statistical incorporation of comonomers into the polymer chain composition; averting reactivity ratio effects and leading to compositionally uniform chains. At higher unreacted monomer concentration, a variety of other parameters become important and the system is termed flooded and at an extreme starts to approach solution or bulk-like reaction conditions.

Starve-fed semi-batch conditions are common in industry and, thus, we searched for a breadth of suitable feed rates that would still satisfy such a condition. While for an acrylate system and without any divinyl crosslinker, Asua et al. [11,13] previously reported that variation in feeding rate did not markedly influence the gel fraction in the final product for *n*-butyl acrylate-seeded emulsion polymerizations, as long as starve-fed conditions were used. This is an important point to emphasize as acrylate-based systems experience a significant amount of chain transfer to polymer producing both short-chain and long-chain branches. The effective mole fraction of labile hydrogens is inherently significant in this case (present in every monomer unit) and, of course, decoupled from monomer feed rate. By contrast, with a methacrylate-based system and no opportunity for hydrogen abstraction, and with a divinyl comonomer, the mole fraction of sites suited to branching and network development (pendent vinyl groups) is a function of crosslinker concentration in the recipe. Both long-chain branching as well as copolymerization with multi-vinyl monomers can lead to gel, and hence we were curious to see if an impact of monomer feed rate would be observed here with a fully methacrylate system (without branching) and specifically with a low mole fraction of divinyl crosslinker.

To establish the tolerance of average concentration of unreacted monomer during these polymerizations as a function of monomer feed rate for the crosslinker containing recipes, a seeded emulsion polymerization with pure *n*-BMA was conducted with a relatively fast feeding time of 30 min. This unreacted monomer concentration in the particles at any point in the reaction is central to this work in that there will be a competitive balance between chain-end propagation and macroradical attack of the pendent vinyl groups present. We are specifically interested in the starve-fed condition and how sensitive that competitive balance is to monomer feed rate variations.

As described in Section 2.3, the monomer feed times for this work range from 0 (batchwise) to 120 min feed and thus the 30 min case is the fastest feed rate (aside from the batch reaction). Characterization of the kinetic profile for this run resulted in a maximum unreacted monomer concentration of 0.7 mol/L and thus safely qualifies as being starve-fed. This of course indicates that all slower feeding rates (longer feed time) would maintain that condition except, of course, for the batchwise reaction. Indeed, the polymerizations containing crosslinker summarized in Table 3 exhibited maximum unreacted monomer concentrations of 0.5 mol/L (30 min feed) down to 0.1 mol/L (120 min feed) allowing a range of four different starve-fed conditions, with the batchwise reaction the only flooded case with an average unreacted monomer concentration of 2.4 mol/L.

### 3.2. Justification of Co-Monomer Selections

While already described to some degree above, the choice of an all-methacrylate system was deliberate for this work. The lack of labile hydrogens for abstraction eliminates alternate pathways to branching and gel formation and for the co-monomer pair of *n*-BMA and EGDMA restricts any topological network formation to reactions with the pendent EGDMA vinyl group. The starved monomer feed condition already forces a statistical incorporation of the monomer mixture in chain composition, but another benefit of the all-methacrylate system is essentially no compositional drift even under flooded conditions. The motivation to produce statistically random copolymers is to remove the potential influence of periods of the polymerization where the average incorporation of the crosslinker is either exacerbated or diminished on the tendency to form gel.

In addition to chemical reactivity motivation, we must also consider the influence of chain mobility on mechanisms and pathways more controlled by diffusion. With a reaction temperature of 70 °C, the glass transition temperature (T_g_) for poly(*n*-BMA-*co*-EGDMA) will always be in the rubbery state (by approximately a 40 °C margin) regardless of unreacted monomer concentration (which will only act to further plasticize the polymer). This low T_g_ will also allow for quicker and more facile swelling by solvent during the analytical gel test and more efficient extraction of linear or lightly branched chains.

### 3.3. Influence of Linear Monomer Feed Rate on Network Formation during the Reaction

Now with a control system basis without crosslinker present, and of course no measured gel content, we explored the aforementioned range of feed times between 0 (batchwise seeded reaction) and 120 min, now with the EGDMA co-monomer to induce gel formation. Table 3 summarizes the major elements of this series of reactions and their gel characteristics. To build comfort in their comparison, we note that all reactions reached high conversion and had consistent control over final particle size (no second crop formation), even for variations in initiator concentration.

Of encouraging note in Table 3 is the trend of gel content sensitivity to the monomer feed rates employed here. It is clear in this type of system that faster monomer feed rates, even under starve-fed conditions, result in higher gel fraction. The batchwise reaction, following trend even though certainly with dramatically higher unreacted monomer content, showed nearly complete gel content by both the solvent extraction technique as well as by A4F. This was not deemed an outlier as the 30 min feed, while still considered starve fed, had a gel content not very different. The discrepancy, while encouragingly small, between the centrifuge analysis and the A4F data can in part be explained by incomplete extraction of linear chains from the uncrosslinked seed polymer for the higher gel content samples and incomplete centrifugation sedimentation for the lower gel-content samples more highly swollen with solvent.

**Figure 1:**
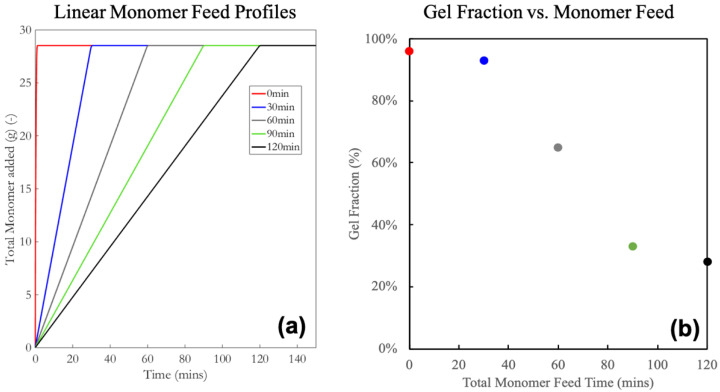
Variations in linear monomer feed rate (**a**) and resulting final gel content (**b**) for equivalent total time at reaction temperature.

A clear trend in Figure 1 is observed that higher linear feeding rate (shorter feed time) led to an increased gel content. It is not surprising that the batch reaction should be able to generate very high gel content as batchwise seeded emulsion polymerization shares similarities mechanistically with crosslinking reactions carried out in bulk or solution copolymerization with EGDMA, which both have often reported for nearly complete gel in their final product [17,18,20]. The trend is clear that topological network development becomes less efficient the slower the monomer is fed to the reactor. That is, with lower unreacted monomer concentration in the reactor the mechanisms that lead to intermolecular, molecular weight increasing, reactions are unfavored. This was somewhat counterintuitive as for example, long-chain branching by chain transfer to polymer reaction pathways are typically more favored with lower unreacted monomer concentration [11,13]. The bottom three rows, coupled with the third row, showcase that for a particular monomer feed rate, the gel fraction result was not strongly sensitive to initiator concentration.

### 3.4. The Impact of Diffusion on Gel Formation Pathways

**Figure 2:**
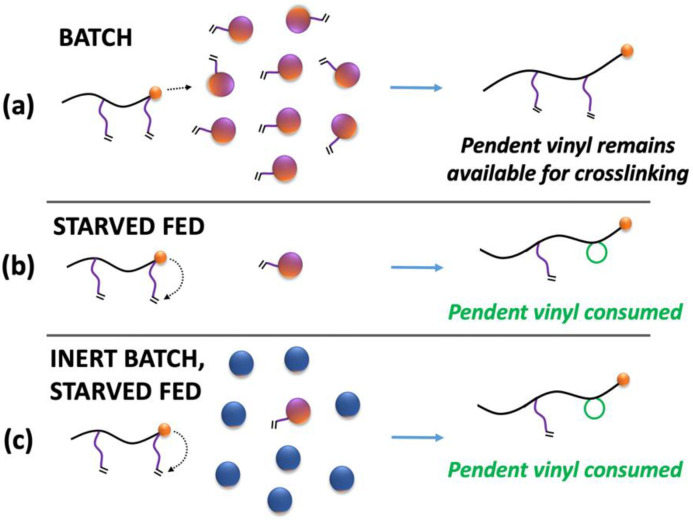
Comparison of probable chain-end radical reaction pathways under high unreacted monomer content (**a**), very low unreacted monomer concentration (**b**), and a hybrid of the two cases where total plasticizer concentration mirrors that of the batch case, but with a reactive fraction similar to that of the starve-fed case (**c**). Orange and blue spheres symbolize plasticizer, where orange spheres with protruding vinyl specifically represent *n*-BMA monomer and blue spheres represent unreactive ethyl acetate molecules. The green microloops are defined later in Scheme 1 and Section 3.5.

Under identical polymerization conditions, a change in the feed time results in differing levels of instantaneous monomer concentrations in the polymer particles. The monomer itself acts as a plasticizer and the amount of residual monomer in the particle significantly impacts the viscosity and the diffusion environment within the particle. The crosslinking reaction involves two macromolecules and is described by mechanisms that require diffusion-related contributions; this ensures the two molecules are within the same reactive local volume, beyond the chemical-controlled reactivity descriptors. This suggests that the plasticizer concentration in the polymer may play a key role in the crosslinking reactions and thus in the resulting gel content. In addition to intermolecular reactive pathways, intramolecular mechanisms such as primary cyclization [21,22,23], are also impacted by chain mobility and diffusion (Figure 2).

To explore this hypothesis, we attempt to isolate and decouple strictly diffusional aspects from chemical reactivity aspects by the addition of an inert plasticizer to an otherwise starved reaction case (Figure 2c). The impact of the plasticizer (monomer or inert solvent) was studied by maintaining similar plasticizer concentration for 2 different feeding times (30 min and 2 h). Both cases significantly differ in final gel content without additional plasticizer (Table 3).

To create similar plasticizer concentrations between both reactions, ethyl acetate (EAc) was used to pre-swell the polymer seed latex in the case of a 2 h feed. EAc was selected due to its similarities with methacrylate monomers [24] and the amount of EAc used was such that the total plasticizer concentration in polymer particles would be comparable to that obtained for the case of the 30 min feed reaction without crosslinker (~0.7 mol/L). This was calculated considering the partitioning of EAc between the polymer and the aqueous phases using a partition coefficient of 3.4 [25]. Based on this the total amount of EAc used was 11.6 wt% (based on total monomer).

From Table 4, we observe that the addition of EAc as plasticizer does not increase the resulting gel content for a 2 h feed, despite the plasticizer concentration being similar to that for 30 min feed. This suggests that the gel formation in the current case is not diffusion-driven and, therefore, points to a different reactive pathway. The amount of reactive monomer in the particles is potentially the key determining factor for the gel formation, as suggested by these experiments. This will be explored further in the next section by utilizing non-linear feeding profiles, where the monomer concentrations differ throughout the reaction.

### 3.5. Seeded Emulsion Polymerization with Non-Linear Feeding

With linear monomer feeding profiles at different rates clearly impacting final gel content, we then turned to explore combinations of different linear feed rates to produce a non-linear overall feed profile. Such profiles are often employed to affect compositional aspects of chains formed in different portions of the reaction or to address engineering issues such as heat removal, yet here we were most curious to discover if the combinations of different linear rates would be additive in cumulative gel or not.

**Figure 3:**
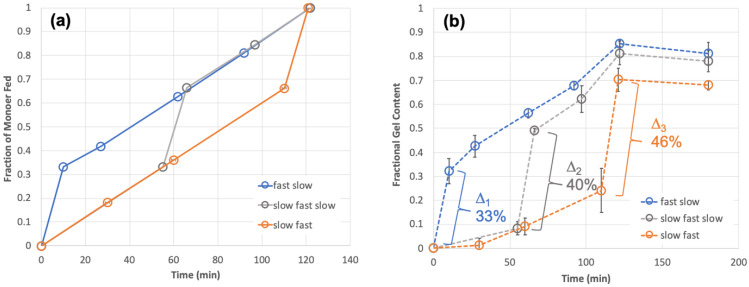
(**a**) Three different non-linear monomer feeding profiles and (**b**) the respective evolution of fractional gel content.

Two linear feeding rates, 10 min feeding time for one third of the total monomer and 110 min feeding time for two thirds of the total monomer, were regarded as “fast feeding” and “slow feeding” rates, respectively. Three different combinations of these two linear feeding rates (fast-slow, slow-fast, and fast-slow-fast) were designed to be the non-linear profiles, as shown in Figure 3a.

From the parent feeding profile (the linear 30 min feed) we observed almost complete gel formation (Table 3), so here, we thought to expect near full gel content for that portion of monomer fed during that stage. Regarding the remaining two thirds of monomer fed by the slow feeding rate, which in fact is even slower than the linear 2 h feeding profile described earlier, we estimated that we might achieve approximately 30% gel for that stage. In the “slow-fast-slow” feeding profile as shown in Figure 3a, each stage contains one third of the total monomer.

The experimental data for these three cases is shown in Figure 3b as fractional gel content instead of instantaneous gel content over reaction time. Fractional gel content can be expressed as the mass of dry gel relative to the product of total monomer mass fed and final monomer conversion. Figure 3b, interestingly, shows that the resulting gel content development profiles displayed similar trends to the monomer feed profiles used. Moreover, while all three non-linear feeding profiles each retained the same total 120 min feeding time, their final gel contents were all observed to be significantly higher (~80%) than the gel content for the corresponding linear 2 h feeding profile (~30%, Table 3). This is an important distinction and even more interesting is that 80% is also significantly higher than the weighted average of each linear feed, which would have been approximately 50% gel.

It was interesting for us to see that different non-linear-feeding profiles can give a very close gel content in the final product, even though the dynamic development of gel content for each was dramatically distinguished. There are two reasons for this. Firstly, gel is formed more easily if there is pre-existing gel in the latex particles; an autoacceleration-like effect. This situation can be confirmed clearly by the gel evolution profile for the “fast-slow” case where almost all monomer fed during the fast period was crosslinked (Δ_1_ = 33%, equivalent to all monomer fed to that point), providing a large existing topological network before the slow feeding profile started. This pre-existing gel means that subsequent chains formed, during the slow feed period, can not only encounter pendent vinyls in network polymer formed during that stage but also encounter pendent vinyls in the pre-existing macromolecular network in the particles. Effectively, once that second feed stage (in this case the slow stage) starts there is a higher overall available pendent vinyl concentration, and a portion is already part of a gel network. Secondly, monomer fed in a later fast feeding profiles could not only crosslink monomer fed in its own period but can also aid in linking prior non-crosslinked polymer together. This effect presents itself more prominently when the amount of non-gel polymer was higher before the fast-feed stage and is clearly reflected by comparing the delta in each profile (Figure 3b) representing the fraction of gel formed specifically during the fast-feeding period. Only one third of monomer was fed in the fast-feeding period in all cases, so any excess gel fraction beyond 33% was contributed by crosslinking reactions with pre-existing non-crosslinked polymer. A result of the same effect, a final observation to highlight in Figure 3b is that at the end of monomer feed (120 min), and similarly after a subsequent one-hour hold time, the earlier the fast feed stage was implemented the higher the observed final gel content.

### 3.6. Hypothesis for Mechanism Responsible for Gel Sensitivity to Monomer Feeding Rate

The heterophase polymerization mechanism of emulsion polymerization brings complexities for the experimental characterization and reasonable interpretation of prevailing mechanisms behind experimental trends observed. Here, we turn our focus to computational efforts to explore mechanisms and in particular to assess the balance of key molecular weight-building pathways sensitive to monomer feeding profile and influencing gel formation.

From the experimental data, it is evident that monomer feeding rate significantly impacts the amount of gel obtained in seeded semi-batch emulsion polymerization. In order to further understand this impact on crosslinking, the emulsion polymerization reactions with EGDMA were simulated using our in-house developed kinetic Monte Carlo (kMC) simulation packages [26,27,28,29]. Previously, our group has successfully utilized kMC methods to simulate crosslinking reactions with various methacrylate based mono and divinyl monomers in bulk polymerization [17,18]. The same approach was utilized here, yet now for reactions in the particle phase while also incorporating aqueous phase reactions and radical entry from the water to the particle phase. The details of this simulation approach for emulsion polymerization are described in Tripathi and Tsavalas [24].

**Figure 4:**
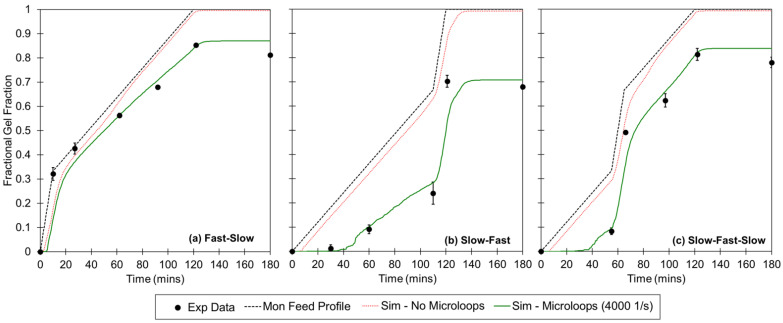
Comparison of experimental (solid points) and simulation results (dashed and solid profiles) for fractional gel fraction versus time for (**a**) fast–slow, (**b**) slow–fast and (**c**) slow–fast–slow non-linear feeding profiles for seeded emulsion polymerization with EGDMA crosslinker (solid green, k_CYC_ = 4000 s^−1^). The monomer feeding profile (dashed black) and simulation without an active micro-loop mechanism (dashed red, k_CYC_ = 0 s^−1^) are also shown for comparison.

The simulation results are shown in Figure 4 (specifically the red short-dashed profiles). Interestingly, we initially found that in all cases regardless of feeding profile, approximately 100% gel fraction was obtained; differing starkly from the experimental observations to this point. This strongly suggested that there are likely to be additional mechanisms involved which rise in importance to dominate in the case of starve fed reactions at low monomer concentration [21,30,31].

**Scheme 1:**
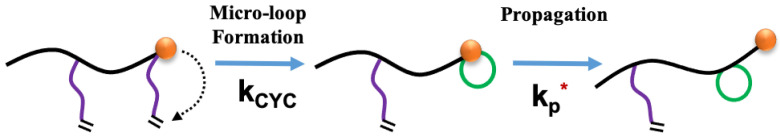
Intramolecular primary cyclization (micro-loop formation) of the active divinyl chain-end radical and continued linear propagation resulting in a consumed pendent vinyl and loss of potential network node.

Crosslinkers like EGDMA have longer ester side chains with a pendent terminal second vinyl group. A radical reaction with that pendent vinyl group (once the first EGDMA vinyl group is part of a growing chain) results in network formation. However, another potential reaction can be considered where a chain-end active radical of a crosslinker’s newly incorporated first vinyl group can potentially react with its own pendent group. This can be seen to be similar to the backbiting reaction prevalent for acrylate monomers [11,13,32,33,34,35] where a chain-end radical abstracts a backbone alpha-hydrogen in close proximity a few monomer units back resulting in a mid-chain radical and short branch. The first-order reaction in crosslinker systems will result in ‘micro-loops’ or intramolecular primary cyclization [21,36,37,38,39,40]. This results in an ineffective network point and does not influence the chain molecular weight, again similar in some ways to short-chain branching from backbiting, yet here this reaction consumes a pendent vinyl group. Much like the 6-carbon ring dimension dominant in backbiting, this micro-loop reaction is also well suited to EGDMA as it develops a loop of similar dimension. This is especially damaging, with crosslinking as the goal, for recipes only involving minor divinyl comonomer fraction. In addition, this reaction event will produce a more entropically restricted mid-chain radical compared to the chain-end radical (Scheme 1, middle) prior to continued propagation. As such, we have modeled this reaction as first-order with a reaction rate coefficient k_cyc_. The subsequent propagation continues at the chain end, yet the first new monomer addition should be noted to be retarded as it will be an addition to a mid-chain radical at the loop closure as opposed to a chain-end radical. This is the reason we inserted an asterisk into the k_p_* on the Scheme as in reality it should be broken into two steps for the retarded first monomer addition and then subsequent propagation forward.

Simulations were performed for a range of values of k_CYC_ and results were compared to the experimental gel data for the non-linear feed systems. From Figure 4 we find that a k_CYC_ value of approximately 4000 s^−1^ results in strikingly close agreement (dark green solid profiles) with the experimental data, even for these more complex multi-stage feed profiles. This is highly encouraging and supportive of the importance of the hypothesis that the probability for the micro-loop pathway versus chain-end propagation is the determining feature for these polymerizations. The rate coefficient estimated for k_CYC_ (~4000 s^−1^) here is higher than that for backbiting reactions observed in *n*-butyl acrylate polymerization which is estimated as 967 s^−1^ [32]. This difference can be attributed to the faster reaction between a chain-end radical and the same monomer’s pendent vinyl group here as compared to that for the chain-end radical intramolecularly abstracting a hydrogen atom further back on the chain backbone.

**Scheme 2:**
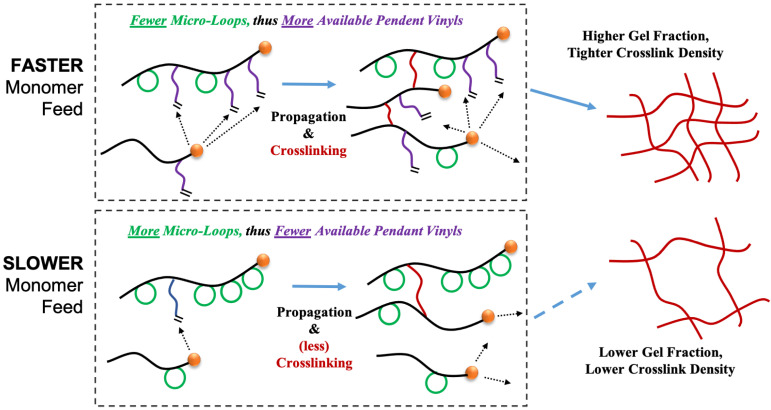
Mechanistic pathways to gel content as a function of monomer feed rate and influenced by propensity of micro-loop formation versus normal chain-end propagation

From the simulations, we find that there is significant retardation in gel formation for the starved system cases as compared to flooded systems where the gel formation is relatively instantaneous. A crosslinker monomer as a chain-end radical has two propagation reaction choices; propagation with another available monomer or intramolecular primary cyclization with itself (Scheme 1). For a flooded system (fast feed systems), the particle contains a large molar concentration of unreacted monomer resulting in higher probability for linear propagation reaction compared to the cyclization reaction. In other words, this will protect and save the pendent vinyl group which can later be attacked by a different macroradical along a crosslinking reaction pathway. On the other hand, for the case of starve-fed systems the molar amount of unreacted monomer in each particle is limited. In such a case, the micro-loop formation reaction will be more comparable to chain-end propagation reactions (depending on crosslinker structural characteristics). This scenario results in higher utilization of the pendent vinyl groups, yet in an ineffective manner removing them from possibility of a network-forming reaction. This also effectively reduces the total molar concentration of pendent groups for any future radical attack. These two scenarios are displayed schematically in Scheme 2.

**Figure 5:**
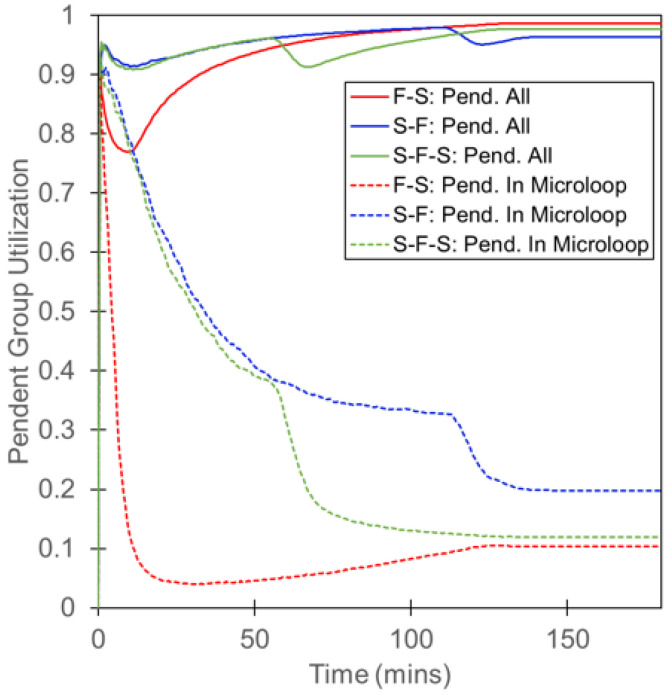
Utilization of pendent vinyl groups on crosslinker monomer. Dashed profiles correspond specifically to those pendent used in microloop formation and solid profiles correspond to total (intra- plus inter-) pendent group utilization. Legend nomenclature for feed profiles indicated by F–S (fast–slow), S–F (slow–fast), and S–F–S (slow–fast–slow).

The impact of feed rate on gel formation can be further discussed in terms of the fraction of pendent vinyl group utilization. In Figure 5, the total fraction of pendent groups utilized (pendent groups reacted relative to amount of crosslinker reacted) is compared with the fraction of pendent groups involved in micro-loop formation for all three non-linear feed systems. Consistent with the experimental gel fraction development, we can see that in the case of the fast fed system, the total pendent group utilization is lower compared the analogous starve-fed condition. Here we can also see that for more monomer flooded (F) segments of the profiles, the ineffective utilization of pendent groups is also reduced. In those segments, the dashed profiles for the fraction of pendent groups utilized in micro-loop formation drop precipitously, retaining those vinyls for potential crosslinking events in subsequent reaction steps. This trend is observed even when the feeding profile is switched to fast feed during later stages of the reaction.

## 4. Conclusions

An exclusively methacrylate-based copolymer of *n*-BMA and EGDMA was polymerized by semi-batch seeded emulsion polymerization to assess the sensitivity of gel network development on monomer feed rate. Methacrylates were chosen so as to avert the complication of parallel pathways to gel from chain transfer to polymer. Strikingly, the gel fraction was observed to be significantly influenced by monomer feed rate, even while most of the experiments were performed under so-called starve-fed conditions. More flooded conditions from faster monomer feed rates, including seeded batch reactions, counterintuitively resulted in significantly higher gel fraction.

We find this counterintuitive as we originally felt there would be some similarity to chain transfer to polymer sensitivity to monomer process conditions which is favored when unreacted monomer content is low. The deviation is in part due to the vastly different mole fraction of reaction sites (i.e., pendent vinyls here versus labile hydrogens in systems that can undergo chain transfer to polymer). Simulations revealed striking correlation to even non-linear monomer feed profiles where the dominant influence on this phenomenon was the sensitivity of primary intramolecular cyclization to the instantaneous unreacted monomer concentration. This is of course directly impacted by monomer feed rate. This is highly encouraging and supportive of the importance of the hypothesis that the probability for the micro-loop pathway versus chain-end propagation is the determining feature for these polymerizations. The rate coefficient estimated for k_CYC_ (~4000 s^−1^) here is also higher than that for backbiting reactions for the case of *n*-butyl acrylate polymerizations which has been estimated as 967 s^−1^ [32]. This difference can be attributed to the faster reactions between a chain-end radical and pendent vinyl groups on a flexible side chain here as compared to that for a chain-end radical abstracting a hydrogen atom from the backbone.

The importance of primary cyclization pathways has been explored previously for bulk and solution polymerizations, but not for emulsified reaction environments with very low mole fraction divinyl monomer where any loss of network-forming nodes becomes dramatically damaging. In addition, while gel fraction could be manipulated by variations in linear monomer feed rates, it could be markedly enhanced by leveraging non-linear feed profiles built from combination sequences of flooded and starved conditions. In this work, we observed 30% gel for a 2 h linear feed time, while 80% gel was observed over the same total feed time when a short fast-feed segment was employed early in the polymerization. This is an important distinction and even more interesting that 80% is also significantly higher than the gel produced by a weighted average of the two corresponding linear feed segments run individually (which would have been approximately 50% gel).

We emphasize here that the ultimate gel fraction in these systems is highly influenced by how early in the polymerization the onset of gel occurs. In bulk polymerizations, this onset occurs considerably earlier under dilute viscosity conditions and very high monomer concentration where, for low mole fraction crosslinker, the micro-loop pathway does not dominate. By contrast, here in seeded emulsion polymerizations the system is viscous from the start where low monomer conditions can enable simpler first order reactions such as intramolecular cyclization, directly impacting the effective mole fraction of subsequent potential crosslinking sites.

An important take-away from this work is that one can maintain starved monomer feed conditions (with unreacted monomer content in polymer particles <1 mol/L), often leveraged in industrial settings for efficient heat removal capability as well as for statistical copolymer composition control, and still have the ability to tune gel content to a desired target. We show here that should one target very low gel content, extension of the monomer feed time is indirectly proportional to gel content observed; longer feed time produces less gel. By contrast, should one target high gel content, non-linear feed profiles with an early fast-feed segment will produce significantly more gel than the equivalent linear feed over the same total time.

## Figures and Tables

**Table 1:** Experimental recipe for the ab initio emulsion polymerization.

Reagent	Mass (g)
*n*-BMA	160.0 ^1^
SDS	3.6
KPS	1.6
Water	559.7

^1^ 10% of the monomer was used as an ab-initio seed followed by the 90% remaining by semi-batch.

**Table 2:** Recipe for the first growth of the initial poly(*n*-BMA) seed.

Reagent	Mass (g)	Additional Water (g)
Seed Latex	172.1	0
*n*-BMA	198.2	0
SDS	1	0
	1	15
	1	15
KPS	1.5	0
Water	512.5	0

**Table 3:** Final products characteristics based on different linear feeding rates.

Feeding Time (hours)	KPS (mol/L)	Conversion (%)	Particle Size (nm) ^a^	Gel Content by Centrifuge (wt%) ^b^	Gel Content by A4F (wt%) ^c^
0	0.01	100%	266	104 ± 4	96
0.5	0.01	99%	264	92 ± 6	93
1	0.01	100%	260	70 ± 10	65
1.5	0.01	98%	263	24 ± 10	33
2	0.01	99%	266	17 ± 5	28
1	0.008	95%	261	60 ± 5	61
1	0.013	97%	262	79 ± 3	76
1	0.03	100%	268	64 ± 3	56

^a^ Particle size was characterized by capillary hydrodynamic fractionation (CHDF), where a ±5 nm error might be considered. ^b^ Gel content was obtained based on the centrifugation method, with uncertainty reported at 95% confidence level. ^c^ Gel Content was obtained by the A4F technique, where an average of ±3wt % error is reasonable.

**Table 4:** Gel content comparison between a 0.5 h fed reaction and solvent pre-swollen reactions with 2 h linear feed profiles.

Feeding Time	wt % ethyl acetate (%) ^a^	Particle Size (nm) ^b^	Gel Content (wt%) ^c^
0.5 h	0	264	92 ± 6
2 h	0	266	17 ± 5
2 h	11.6	268	12 ± 10

^a^ Weight percent inert solvent based on monomer during crosslinking stage. ^b^ Particle size was characterized by CHDF, where a ±5 nm error is reasonable to consider. ^c^ Gel content was obtained based on the centrifugation method.
